# Infection‐Triggered Disease Flare With Extraintestinal Manifestations in Trichohepatoenteric Syndrome: A Case Report

**DOI:** 10.1155/carm/3024414

**Published:** 2026-05-31

**Authors:** Hatem M. Taha, Mohammed AbuBaha, Hossam Salameh, Wael Hashem, Amin A. A. Abualhommos, Omar Marouf, Hasan Khalili

**Affiliations:** ^1^ Head of Medical Departments, Palestine Medical Complex, Ramallah, State of Palestine; ^2^ Department of Medicine, An-Najah National University, Nablus, State of Palestine, najah.edu; ^3^ Department of Internal Medicine, Palestine Medical Complex, Ramallah, State of Palestine

**Keywords:** immunodeficiency, intractable diarrhea, osteomyelitis, *SKIC3* gene, trichohepatoenteric syndrome, *TTC37* gene

## Abstract

Trichohepatoenteric syndrome (THES) is a rare, autosomal recessive disorder characterized by early‐onset diarrhea, woolly hair, and facial dysmorphism and variable multisystem involvement. Herein, we report a 17‐year‐old male with a genetically confirmed THES who presented with worsening diarrhea, fatigability, weight loss, and epigastric pain following intestinal amebiasis. His disease course was notable for extraintestinal and inflammatory manifestations, including chronic recurrent multifocal osteomyelitis, syringomyelia, and inflammatory bowel disease–such as enteropathy, highlighting a broad phenotype. The patient had intractable diarrhea since the age of 2, positive antiendomysial antibodies with no histologic confirmation, and a poor response to standard inflammatory bowel disease therapy. This case highlights the potential role of infectious triggers in precipitating disease flares in THES and demonstrates the diagnostic challenges posed by overlapping clinical and serological features with other autoimmune and inflammatory conditions. Our study broadens the clinical spectrum of THES and emphasizes the importance of detecting infection‐related exacerbations and extraintestinal symptoms in adolescent survivors.

## 1. Introduction

Trichohepatoenteric syndrome (THES) or syndromic diarrhea (SD) is a rare autosomal recessive disorder with multisystemic manifestations starting early as unexplained neonatal‐onset diarrhea [1].

According to the literature, the incidence rate of THES is estimated to be 1:1,000,000 in France and 1:200,000 in Saudi Arabia, which can be attributed to consanguineous marriages. This is further supported by the observation that most patients in the French cohort were originally from Arab regions where consanguinity rates are elevated [2].

In general, clinical manifestations are quite similar with minor variations from one case to another, but certain clinical signs that have been proven to be evident in almost all cases include intractable diarrhea, woolly uncombable hair, and facial dysmorphism [1, 3], though in 2019, a reported case from Australia confirmed a diagnosis of THES without diarrhea which has not been reported before [4].

Other features that may also be evident include intrauterine growth restriction (IUGR) and immunodeficiency, with less evident features such as skin pigmentation and liver disorders, and two manifestations that occur infrequently are congenital cardiac defects and platelet abnormalities [1–3].

THES is a multisystemic disease classified under the umbrella of enterocyte epithelial alteration disorders, which also includes microvillus inclusion disease and tufting enteropathy [1]. The diagnosis is confirmed by genetic testing demonstrating pathogenic variants in either *SKIC3* or *SKIC2*, previously known as *TTC37* and SKIV2L, respectively [1]. Here, we report an adolescent male with genetically confirmed THES who experienced an infection‐triggered disease flare accompanied by unique extraintestinal manifestations, including recurrent multifocal osteomyelitis and syringomyelia.

## 2. Case Presentation

A 17‐year‐old male, a known case of THES diagnosed at the age of 2, was admitted to our hospital with a one‐week history of generalized weakness and fatigue. He reported a decreased oral intake due to colicky epigastric pain, lasting approximately 1 hour daily over the past week. The pain worsened with food intake and was partially relieved by analgesics and defecation. Additional complaints included daily vomiting and diarrhea, with six to seven loose stools per day. The diarrhea was brownish in color, without mucus, and not foul‐smelling. Four days prior to admission, he presented to a local emergency room, where stool analysis revealed amebiasis; thus, he was managed with intravenous fluids and was discharged with a prescription for metronidazole and potassium chloride; however, he showed minimal clinical improvement.

The patient’s family history is notable for parental first‐cousin consanguinity. He is the third of four siblings; while his younger brother is healthy, his two older sisters have also been diagnosed with THES. The patient’s clinical journey began at the age of 2, when he developed an intractable diarrhea, progressive abdominal distention, and periumbilical pain relieved by defecation. Initial workup revealed positive antiendomysial antibodies with normal total IgA levels; however, no diagnosis of celiac disease was established. Barium enema, stool analysis, and stool pH (6.0) were all unremarkable. Given the positive family history and clinical presentation, THES was highly suspected. Therefore, genetic testing with Sanger sequencing was performed, which identified a homozygous variant: c.2282T > C (p.Leu761Pro) in the *TTC37* gene.

At the age of six, the patient experienced a clinical exacerbation of diarrhea and abdominal distention, for which an upper endoscopy was done, which revealed diffuse gastric erythema and ulcerations along with exudates in the duodenal bulb. Further evaluation with colonoscopy showed an inflamed, dilated colon, raising suspicion for inflammatory bowel disease (IBD); however, no biopsies were taken to confirm. Cystic fibrosis was also suspected, yet a sweat chloride test was 12 mmol/L (normal < 30 mmol/L). Nevertheless, pancreatic enzyme replacement therapy (Creon) and zinc supplementation were initiated.

**FIGURE 1:**
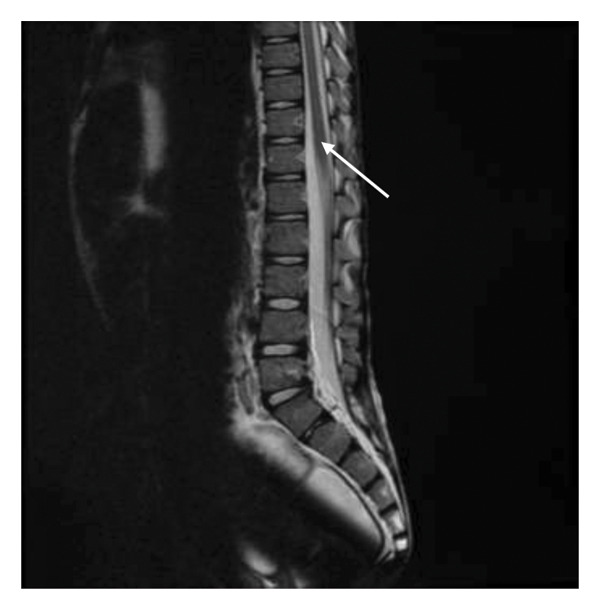
Sagittal T2‐weighted MRI shows a hyperintense fluid‐filled syrinx within the thoracic spinal cord.

Throughout his childhood, the patient also experienced an abnormal gait and frequently complained of bilateral knee pain while walking. While knee radiographs at age 12 were unremarkable, a spiral MRI revealed an abnormal longitudinal high signal within the spinal cord, consistent with syrinx (Figure 1). A subsequent brain MRI ruled out a suspected Chiari malformation. He was later followed up at a separate medical institution, where he was diagnosed with chronic recurrent multifocal osteomyelitis (CRMO) and was therefore started on bisphosphonates, methotrexate, and prednisone.

Upon his current admission at age 17, initial laboratory evaluations (Table 1) were performed to guide management. He was managed with multiple medications since admission, including analgesics such as paracetamol and intravenous lidocaine, antiemetics such as metoclopramide, gastrointestinal protectants such as pantoprazole, antibiotics including metronidazole and ciprofloxacin, corticosteroids such as dexamethasone and hydrocortisone, potassium chloride and magnesium sulfate for electrolyte management, and intravenous fluids as needed. His clinical condition has gradually improved throughout the hospitalization, and he was discharged on Day 4. Regular outpatient follow‐up evaluations to monitor his laboratory parameters and clinical status are required.

**TABLE 1:** Initial laboratory data during the latest admission.

Test parameter	Normal range	Result
Hemoglobin	13.5–17.5 g/dL	8
Hematocrit	43.5%–53.7%	24.7
Mean corpuscular volume	80–100 μm^3^	64.3
Mean corpuscular hemoglobin	27–31.2 pg/cell	20.8
Mean corpuscular hemoglobin concentration	31%–35% Hb/cell	32.3
RBC count	4.6–6.1 million/mm^3^	3.84
WBC count	4.6–11 × 10^9^/L	10.6
Platelet count	140–450 × 10^9^/L	588
C‐reactive protein	< 0.3 mg/dL	14.9
Calcium	8.4–10.2 mg/dL	7.28
Magnesium	1.6–2.6 mg/dL	2.43

Table 2 summarizes the clinical course of the patient.

**TABLE 2:** Clinical timeline.

Age/period	Clinical event	Investigations	Diagnosis/interpretation	Management
Infancy (2 years)	Onset of chronic watery diarrhea, abdominal distention, and periumbilical pain.	Positive antiendomysial antibodies (normal IgA); normal barium enema and stool analysis (pH 6).	Suspected trichohepatoenteric syndrome (THES).	Supportive care and initial diagnostic evaluation.
Early childhood	Persistence of enteropathy	Sanger sequencing: Identified homozygous variant c.2282T > C (p.Leu761Pro) in the TTC37 gene.	Confirmed THES.	Nutritional optimization and symptomatic relief.
Childhood (6 years)	Exacerbation of diarrhea and abdominal distention.	Upper endoscopy: Gastric erythema/ulcers and duodenal exudates. Colonoscopy: Colonic dilation and inflammation.	Suggestive of inflammatory bowel disease (IBD) overlap; cystic fibrosis excluded (sweat chloride: 12 mmol/L).	Pancreatic enzyme replacement (Creon) and zinc supplementation.
Adolescence (12‐16 years)	Abnormal gait, chronic bilateral knee pain, and refractory diarrhea.	Spiral MRI: Longitudinal high‐signal cord lesion (syrinx). Imaging and inflammatory markers: For bone pain.	1. Syringomyelia (neurologic THES involvement). 2. Chronic recurrent multifocal osteomyelitis (CRMO).	Bisphosphonates, methotrexate, and prednisone; nutritional modulation.
Current presentation (17 years)	Weakness, fatigue, and colicky epigastric pain.	Stool analysis positive for *Entamoeba histolytica*; abnormal inflammatory markers and electrolytes	Acute infectious exacerbation (amebiasis) on background of THES.	IV fluids, metronidazole, ciprofloxacin, and electrolyte correction.
Hospital course	Gradual resolution of gastrointestinal and systemic symptoms.	Continuous clinical and laboratory monitoring.		Discharged on Day 4 with close outpatient follow‐up.

## 3. Discussion

THES, a rare autosomal recessive disease, is characterized by a constellation of symptoms involving early onset intractable diarrhea, woolly hair, IUGR, and facial dysmorphism [1]. Other features include immune dysfunction, recurrent infections, and hepatic involvement (Table 3) [1]. It was first described by Stankler in 1982 [2]. The underlying genetic component was later found to be linked to mutations in *SKIC2* (formerly *SKIV2L*) and *SKIC3* (formerly *TTC37*) genes, both of which are involved in the human Ski complex that plays a role in RNA exosome‐dependent RNA degradation and regulation pathway [1, 5].

**TABLE 3:** Clinical manifestation of trichohepatoenteric syndrome.

System involved	Description		Prevalence (%)^∗^	Present in this case (yes/no)
Gastrointestinal	Intractable diarrhea	Neonatal onset of chronic diarrhea despite fasting	≈100 [1]	Yes
	Colitis, gastritis	Inflammatory disease of the GI tract	≈73 [1]	Yes

Skin and musculoskeletal	Woolly hair	Uncombable, easily removable, fizzy hair, usually with evidence of trichorrhexis nodosa	≈100 [1]	Yes
	Facial dysmorphism	Wide forehead, broad nasal root, hypertelorism, and coarse facial features	≈100 [1]	No
	Skin changes	Café au lait, xerosis, rubbery skin	≈50 [1]	No

Immune system	Defective immune responses	Low IgG level, defected AB response after vaccination, low lymphocyte count	≈88 [1]	No
IUGR/SGA	Intrauterine growth restriction, small for gestational age	Below the 10th percentile for gestational age, preterm delivery	≈67 [1]	No
Liver disease	Various liver pathologies	Cirrhosis, siderosis, isolated hepatomegaly	≈52 [1]	No
Cardiac abnormalities	Various cardiac pathologies	Aortic insufficiency, peripheral pulmonary stenosis, tetralogy of Fallot, ASD/VSD, patent ductus arteriosus	≈25 [1]	No

^∗^Prevalence estimates are based on the GeneReviews 2018 (1) compilation, and the figures reported may evolve as additional cohort data emerge.

Our patient, with a genetically confirmed diagnosis of THES in infancy, presented with an acute exacerbation of generalized fatigue, chronic diarrhea, and significant weight loss. These symptoms occurred concurrently with a documented amebic infection, raising the possibility that intestinal infections may precipitate systemic decompensation in THES. Despite a handful of studies reporting severe infections coinciding with THES, there was no standardized definition of THES episode in these papers. And thus, to our knowledge, infection‐triggered disease flares have not been explicitly reported in prior THES case series or in closely related monogenic enteropathies; this observation should therefore be regarded as a hypothesis‐generating finding rather than an established association. A plausible mechanistic basis involves immune dysfunction such as hypogammaglobulinemia [6], combined with an impaired mucosal barrier secondary to villus atrophy and chronic enteropathy. These factors increase intestinal permeability and susceptibility to microbial translocation, potentially amplifying systemic inflammation and contributing to multisystem vulnerabilities [1, 7].

In addition to intestinal symptoms, our patient exhibited several extraintestinal symptoms, including gait abnormalities, chronic arthralgia, and CRMO, while these symptoms have not been classically described in THES. A plausible association can be hypothesized through shared immune dysregulation as follows: CRMO is an autoinflammatory, nonbacterial bone disease caused by defects in innate immune signaling [2]. It is strongly associated with other immune‐mediated conditions such as IBD and psoriasis [3, 4]. This suggests a background of systemic immune dysfunction rather than bone‐limited pathology [2]. Moreover, the potential link with intestinal dysbiosis and chronic gut inflammation has been discussed and hypothesized in the literature [2, 5]. Even though there are no studies that explicitly describe CRMO in individuals with THES, one can speculate that the combination of gastrointestinal and immune disturbances in THES might predispose to similar innate immune activation, creating a biological plausible basis for CRMO to happen.

Regarding syringomyelia, there is currently no published evidence linking it to THES. Any association between the two conditions must therefore be considered hypothetical; one could speculate that long‐standing inflammation and malnutrition might contribute to spinal cord pathology. The most prudent interpretation based on available data is that our patient has two independent conditions, unless future reports demonstrate a reproducible association. An interesting aspect in our patient’s differential was celiac disease, as evidenced by the positive antiendomysial antibodies; this can add to the complexity of the diagnostic process, potentially delaying diagnosis or putting clinicians at risk of misdiagnosis, as THES symptoms can mimic celiac disease. This overlap has been mentioned before in other case reports [8]. However, persistence of symptoms despite following a gluten‐free diet and the overall clinical picture were more consistent with THES.

The relationship between THES and IBD, particularly very early onset IBD (VEOIBD), is of particular interest due to the clinical overlap. According to the medical literature, patients with THES can present with GI manifestations closely mimicking IBD, such as chronic diarrhea, colitis, ileitis, panenteritis, and perianal disease [9]. In fact, THES is a recognized monogenic form of IBD [6, 7].

Histopathological and clinical findings may also resemble those seen in IBD cases, and laboratory indicators such as elevated fecal calprotectin and ESR have been identified in some cases, further supporting the IBD‐like phenotype in THES [9]. However, pathogenesis and therapeutic responses between IBD and THES differ significantly [9]. THES is caused by mutations affecting RNA exosome complex components, leading to a monogenic condition with a different molecular etiology compared to IBD [9].

Notably, standard IBD therapies such as mesalazine, corticosteroids, and immunomodulators have shown modest or transient efficacy in THES patients with IBD‐like features [9]. This poor response further highlights the unique pathophysiology of THES‐associated enteropathy and implies that the inflammatory process in THES is not driven by the same mechanism as in IBD [9]. This is further supported by published case series; one study of six patients with THES reported partial or no response to standard IBD therapies [8]. Another study found that steroids and immunosuppressants showed little to no efficacy with only transient or partial improvement in some IBD‐like cases [9]. A UK‐based cohort also reported that 5‐aminosalicylates were ineffective and conventional immunosuppressive therapies were of a limited effect [10].

This patient’s prolonged history of arthralgia, abnormal gait, and syrinx formation on spinal MRI, with subsequent treatment with corticosteroids, bisphosphonates, and methotrexate further supports the existence of an underlying systemic inflammatory process.

In terms of management, it remains supportive, including enteral feeding, parenteral nutrition, and organ‐targeted treatment [7]. One of the objectives to be achieved is to promote patients’ appropriate weight gain, reduce the burden of infections, and individual management of intellectual disability [1]. Immunoglobulin supplementation can also be used for patients with low immunoglobulin levels and has been shown to decrease the infection rate among THES patients [1]. This case contributes to the literature that THES encompasses a wide phenotypic spectrum, including immune‐mediated bone disease, neurological abnormalities, along with classical features, and points out the role of infectious triggers in precipitating disease flares. A recent case series by Ozturk et al. [11] further expanded the phenotypic and genotypic repertoire of THES, documenting previously unreported findings such as osteoporosis, radioulnar synostosis, and nephropathy across eight patients from five unrelated families, underscoring the breadth of atypical manifestations that clinicians should be alert to [11]. Recognizing and understanding these associations can help improve diagnostic accuracy and coordinate long‐term management, especially in places where consanguinity rates are high, as they increase the risk of acquiring this autosomal recessive disorder; therefore, especially in Palestine, THES should be kept in mind when thinking of the differential.

## 4. Limitations

Although serum IgA was within normal limits, extended immunological investigations, including quantitative IgM and IgG levels, and flow cytometric immunophenotyping, were not available for this patient.

## Funding

No funding was received for this manuscript.

## Ethics Statement

All procedures performed in this report involving human participants were in accordance with the ethical standards of the institutional, national research committee, and with the 1964 Helsinki declaration and its later amendments or comparable ethical standards.

The authors confirm that all mandatory laboratory health and safety procedures have been complied within the course of conducting any experimental work reported in this paper.

## Consent

The authors obtained verbal and written informed consent from the patient regarding this case and any accompanying images. A copy of the written consent is available for review by the Editor‐in‐Chief of this journal on request.

## Conflicts of Interest

The authors declare no conflicts of interest.

## Data Availability

The data that support the findings of this study are available from the corresponding author upon reasonable request.
